# Factors associated with past 30-day abstinence from cigarette smoking in adult established smokers who used a JUUL vaporizer for 6 months

**DOI:** 10.1186/s12954-019-0331-5

**Published:** 2019-11-07

**Authors:** Christopher Russell, Farhana Haseen, Neil McKeganey

**Affiliations:** Centre for Substance Use Research, 4.04 West of Scotland Science Park, 2317 Maryhill Road, Glasgow, G20 0SP UK

**Keywords:** E-cigarettes, Vaping, Quitting, Smoking, Cigarettes, Tobacco harm reduction

## Abstract

**Background:**

JUUL is the fastest growing and highest selling brand of e-cigarette/vapor products in the USA. Assessing the effect of JUUL vapor products on adult smokers’ use of conventional tobacco cigarettes can help inform the potential population health impact of these products.

**Methods:**

Participants were 15,456 US adult established current smokers aged 21 years who had purchased their first JUUL Starter Kit from a retail store or online within the past 7 days. Online surveys assessed past 30-day use of conventional cigarettes, JUUL vapor products, and other e-cigarettes/vapor products at 3 and 6 months after their first JUUL purchase. Logistic regression models examined factors associated with smokers’ odds of self-reporting past 30-day abstinence from cigarette smoking at 6 months.

**Results:**

Past 30-day point prevalence abstinence from smoking at 6 months was 31.6% in the intent-to-treat (ITT) sample and 54.0% among those who responded at 6 months (*n* = 9040; 58.5% of ITT). Consecutive past 30-day smoking abstinence outcomes at 3 and 6 months were reported by 20.3% of the ITT sample and 40.6% of responders to both assessments (*n* = 7726). Covariate-adjusted odds for reporting past 30-day smoking abstinence at 6 months were significantly higher among primary users of mint- or mango-flavored JUULpods (compared to primary users of Virginia tobacco-flavored JUULpods), exclusive users of JUULpods in characterizing flavors (compared to exclusive users of tobacco-flavored JUULpods), daily users of the JUUL vaporizer (compared to less-than-daily), initial retail purchasers (compared to initial e-commerce purchasers), and those who first purchased a JUUL to help to quit smoking completely. Odds for reporting past 30-day smoking abstinence were significantly lower among those who, at study enrolment, had smoked regularly for ≥ 20 years, smoked ≥ 10 cigarettes per day, and smoked on all 30 of the previous 30 days.

**Conclusions:**

Around one third of enrolled smokers and one half of smokers who responded to a 6-month follow-up reported being past 30-day abstinent from cigarette smoking after using a JUUL vaporizer for 6 months. More frequent use of a JUUL vaporizer and primary use of JUULpods in characterizing flavors, particularly mint and mango, appeared to be important to smokers’ chances of quitting. The impact of suspending retail sales of flavored JUULpods on adult smokers’ likelihood of quitting should be closely assessed.

## Background

Smoking cigarettes and other combusted tobacco products continues to kill more people, cause more disease, and contribute more to health inequalities in high-income countries than any other preventable factor [1]. In the USA, 480,000 Americans die annually from smoking-related diseases, and around 16 million American adults are currently living and suffering with a smoking-related disease [2, 3]. On average, smokers lose around 3 months of life for every year of smoking after age 35, equating to around 10 years of life lost by lifelong smokers compared to non-smokers. Quitting tobacco smoking at the soonest opportunity is therefore the best action a person can take to reduce risks for developing chronic illness and increasing life expectancy [4, 5]. If global cigarette sales continue on trend through to 2030, around 8 million people are projected to die prematurely from a smoking-related disease each year, the majority of whom will be people who are currently smoking, not those who have yet to start [6]. Therefore, while smoking prevention efforts are critically important, encouraging and supporting more smokers to attempt to quit and providing smokers with the support and means they need to have the best chance of succeeding in their quit attempt will have a greater impact on population mortality and morbidity in the short term.

Quitting smoking is notoriously difficult, with only 3–5% of those who quit without assistance and fewer than 10% of all smokers achieving long-term abstinence, and often only after many unsuccessful quit attempts [7, 8]. Though not approved by the US Food and Drug Administration as an aid to smoking cessation, electronic cigarettes (e-cigarettes)—hand-held devices that use battery power to heat a solution of propylene glycol, glycerol, and often flavorings and nicotine, to produce an aerosol that the user inhales—have become the most popular assisted method of quitting smoking in the USA, used in 35% of smokers’ most recent quit attempts [9]. Though inconclusive, evidence from multiple population-based repeated cross-sectional [10–16] and longitudinal observational studies [17, 18] and from randomized controlled trials [19, 20] suggests that using e-cigarettes as part of a quit attempt can aid cessation. Data from multiple years of the US Current Population Survey-Tobacco Use Supplement (CPS-TUS), for example, indicate the substantial increase in e-cigarette use that occurred between 2010 and 2015 was associated with a statistically significantly 1.1% increase in the rate of smoking cessation at the population level during these years [10], with smokers who used e-cigarettes in 2014–2015 more likely to have attempted to quit smoking (65.1% vs. 40.1%) and more likely to have succeeded in quitting smoking for at least 3 months (8.2% vs. 4.8%) compared to non-users of e-cigarettes. This 1.1% increase in the smoking cessation rate, equivalent to approximately 350,000 additional ex-smokers, was the first significant increase in the smoking cessation rate at the population level in the USA for the past 25 years.

Other longitudinal research, however, has suggested regular e-cigarette use is associated with an increased likelihood of attempting to quit and with a substantial reduction in cigarette consumption, but not with smoking cessation at a 6-month follow-up [21] or 1-year follow-up [22], and that frequent or daily e-cigarette use is associated with higher odds of quitting smoking [23–25], but also with a significantly higher probability of relapse to smoking among men [26]. Additionally, though most adults who completely switch from cigarette to e-cigarettes report e-cigarettes to be a satisfying alternative to cigarettes, around five times more current smokers who tried using e-cigarettes rated them as less satisfying than cigarette and stopped using them, suggesting that, for many current smokers, e-cigarettes will have to become much more satisfying if they are to replace cigarettes [27].

A common feature of these studies’ assessment of the potential cessation efficacy of e-cigarettes, however, was their consideration of e-cigarette as a homogeneous category of products. The population’s use and impacts of e-cigarette use, however, are likely to vary across an increasing number and heterogeneity of e-cigarette devices (e.g., size, shape, voltage), e-liquids (e.g., flavors, quality, constituents), and use behaviors (e.g., frequency of use in a month/day, puff duration, puff frequency). Obtaining data that characterize the efficacy of specific brands, makes, and models of e-cigarettes for aiding smoking cessation in real-world and trial settings is therefore key to understanding the potential contribution of each individual e-cigarette product to future population health.

The JUUL brand of vaping products has become the fastest and highest selling brand in the US e-cigarette market [28]. The JUUL vaporizer is a pod-based e-cigarette that is based on a two-part system: a pre-filled, disposable e-liquid pod that clicks into a small battery. A prospective study of adult established current smokers making a first-time purchase of a JUUL vaporizer found that, 3 months later, 28.3% reported having not smoked any cigarettes for at least 30 days, with the rate of quitting found to be significantly higher among daily users of the JUUL vaporizer and among those who primarily used a JUUL vaporizer containing mint- and mango-flavored pods [29]. Longer-term data are needed, however, in order to characterize the rate at which 3-month quitters continue to report quitting across further follow-ups, the rate at which those who were still smoking at 3 months subsequently report quitting, and the rate at which 3-month quitters report having lapsed back to smoking at further follow-ups.

In this study, we analyzed data from an ongoing prospective study of changes in smoking behavior in a large cohort of adult established current smokers in the USA up to 6 months after their first purchase of a JUUL vaporizer. We examined the rates at which new JUUL users reported having quit smoking completely or continued to use both cigarettes and the JUUL vaporizer after 3 and 6 months. We also report the rates at which those new JUUL users who had quit smoking after 3 months (i) maintained this quit or (ii) had resumed smoking at the 6-month follow-up, and the rates at which new JUUL users who reported using both cigarettes and the JUUL vaporizer at 3 months (iii) had quit smoking completely or (iv) continued to use both products at the 6-month follow-up. Additionally, we examined the extent to which demographic, smoking, and e-cigarette-related factors predicted higher and lower odds of new JUUL users reporting having quit smoking completely at the 6-month follow-up.

## Methods

### Sample and recruitment

Eligible individuals were US adults aged 21 years and older who had smoked at least 100 cigarettes in their lifetime, now smoke cigarettes “every day” or on “some days,” and had purchased their first JUUL vaporizer Starter Kit from a US retail store or through JUUL Labs Inc.’s e-commerce store at www.juulvapor.com within the past 7 days. Individuals were eligible to participate whether or not they intended to use a JUUL as an aid to quitting smoking. Eligibility in response to each criterion was self-reported, with the exception of being aged 21 years or over, which was verified by *Veratad Technologies’* age verification software, *AgeMatch*^*SM*^ at the point of an attempted online purchase. A JUUL Starter Kit contains a JUUL vaporizer, a USB charging dock, and one pod in each of four flavors (1× Virginia tobacco, 1× mint, 1× mango, and 1× crème). Each e-liquid pod sold in a JUUL Starter Kit contains 0.7 mL e-liquid and is designed to contain 41 mg of nicotine (59 mg/ml nicotine).

Individuals were invited to participate in this study in two ways. First, JUUL Labs Inc. sent email invitations to 37,536 age-verified adults who had purchased a JUUL vaporizer Starter Kit through JUUL’s e-commerce store between 4 April 2018 and 25 June 2018. The email invited individuals to participate in a 6-month online survey study about their use of combustible cigarettes, JUUL vaping products, and other brands of e-cigarettes and vaping products. Invitations were sent to the email address associated with a customer’s age-verified account. An email invitation containing the web-link to the baseline survey was scheduled to be sent to each individual approximately 4 days after they completed their online purchase of a JUUL Starter Kit so as to be received by the individual within 48 h after the scheduled delivery of their purchased product(s).

Second, individuals who purchased a JUUL vaporizer Starter Kit in a retail store were invited to participate via 3″ × 2.5″ cards that were manually inserted into the packaging of 500,000 JUUL vaporizer Starter Kits, which were then distributed at random to approximately 10,000 licensed store retailers of JUUL vaporizer products across the USA. Starter Kits containing invitation cards were distributed across April 2018. The invitation cards contained within these Starter Kits were used to recruit new JUUL purchasers to several research studies in addition to the present study. It is not known how many of the Starter Kits containing invitation cards remain unsold on store shelves.

Printed on each invitation card insert were the invitation text, the survey web address, and a unique six-digit alphanumeric code. Individuals who purchased a JUUL vaporizer Starter Kit that contained an invitation card insert were invited to type the survey web address—survey.juul.com—into their web browser, and then, when prompted, type the six-digit code displayed on their invitation card insert. Entry of a valid code routed the individual to an Account Creation webpage, and then to the study Informed Consent Form. Each six-digit code was valid for one entry; attempts to re-use the code were blocked. Requiring the entry of a unique, one-time access code ensured that only individuals who had purchased a JUUL vaporizer Starter Kit in a retail store could proceed to the Account Creation webpage, and requiring individuals to create a user account ensured that only one survey could be completed per account.

### Procedure

The first page of the survey displayed an Informed Consent Form (available upon request), which described the purpose of the survey, the names and contact details of the study investigators, information about who is eligible to take part and how survey data will be used, assurances of participant anonymity and confidentiality, and the source of funding for this study. Participants were informed they were being invited to take part in six monthly online surveys about their use of combustible cigarettes, JUUL vaporizer products, and other e-cigarettes and vapor products. Individuals who satisfied eligibility criteria and gave informed consent to participate began the survey. Participants were routed to questions that were applicable to them on the basis of a response or combination of responses to a previous question or questions. The survey instrument was designed with the assumption that all respondents to a question would be asked the next question, unless there were specific instructions routing a subgroup of respondents to a different question. Participants answered survey questions at their own pace. If a participant did not complete the survey, all data provided up to the point of exit from the survey was excluded from analysis.

The baseline survey took around 15 min to complete. Participants who completed the baseline survey received an automated email invitation to complete a follow-up survey 30 ± 5 days, 60 ± 5 days, 90 ± 5 days, and 180 ± 5 days after completion of the baseline survey. Two reminder emails were sent to non-respondents within each 10-day window. Participants received a USD$30 virtual Visa Reward Card by email for each survey they completed.

### Measures

#### Cigarette smoking in the past 30 days

The primary outcome measure in this study was self-reported past 30-day abstinence from cigarette smoking, which was determined at each assessment by a “No” response to the question, “In the past 30 days, have you smoked a cigarette, even one or two puffs?” Participants who indicated they have smoked a cigarette in the past 30 days were asked two further questions about their frequency of smoking in the past 30 days—“Do you now smoke cigarettes…” (every day, some days, not at all), and “On how many of the past 30 days did you smoke cigarettes?” (numeric response, 1–30), and one question about their intensity of smoking in the past 30 days—“On those days that you did smoke, how many cigarettes did you usually smoke each day? A pack usually has 20 cigarettes in it”. Participants who did not provide answers to these four questions at the baseline assessment were excluded from the analytic sample.

#### Cigarette smoking history

Questions assessed the age at which participants first smoked a cigarette, first started smoking regularly, the number of months/years for which participants had been smoking cigarettes regularly, and the number of cigarettes participants had smoked in their lifetime.

#### Use of a JUUL vaporizer and JUULpod flavors in the past 30 days

Questions assessed the number of days in the past 30 days on which participants had used a JUUL vaporizer and the total number of JUUL vaporizer refill pods they had consumed in each of eight commercially available flavors (Virginia tobacco, mint, mango, crème, fruit, cucumber, classic tobacco, and menthol) in the past 30 days. Participants were coded as a “primary user” of a specific flavor of JUULpods when they reported having consumed more pods in that flavor than in any other flavor. For example, a participant who reported having consumed 10 mango-flavored JUULpods and 5 mint-flavored JUULpods in the past 30 days would be coded as a primary user of mango-flavored JUULpods.

Participants were coded as “past 30-day exclusive users of tobacco flavors” if they reported use of only Virginia tobacco- and/or classic tobacco-flavored JUULpods in the past 30 days. Participants were coded as “past 30-day exclusive users of characterizing flavors” if they reported use of only mint-, mango-, crème-, fruit-, cucumber-, and/or menthol-flavored JUULpods in the past 30 days. Participants were coded as “past 30-day users of both tobacco and characterizing flavors” if they reported consumption of at least one pod in Virginia tobacco or classic tobacco flavor and at least one pod in mint, mango, crème, fruit, cucumber, or menthol flavor.

#### Use of e-cigarettes other than a JUUL vaporizer in the past 30 days

Participants were asked if they had used any brand of e-cigarette or vaping device other than a JUUL vaporizer in the 30 days prior to each assessment.

#### Reasons for purchasing and using a JUUL vaporizer Starter Kit

At the baseline assessment, participants were asked to identify which, if any, of a list of health, social, financial, sensory, and convenience reasons were reasons why they first decided to purchase a JUUL Starter Kit.

#### Demographics

Questions assessed sex, age, race/ethnicity, educational attainment, annual household income, and census region of participants’ residence.

### Data analysis

Rates of past 30-day abstinence from smoking at the 3-month and 6-month follow-up assessments are reported for the intention-to-treat (ITT) sample (*N* = 15,456) that completed the baseline assessment. At each follow-up assessment, participants with a missing response to the question “In the past 30 days, have you smoked a cigarette, even one or two puffs?” were recoded as “smoked in the past 30 days” under the worst-case scenario assumption that these participants had returned to baseline patterns of cigarette smoking.

Rates of past 30-day abstinence from smoking at the 3-month and 6-month follow-up assessment are also reported for efficacy subsets of participants who provided smoking data at the 3-month assessment (*n* = 9272, 60.0% of the ITT sample) and the 6-month assessment (*n* = 9040; 58.5% of the ITT sample), respectively. A rate of past 30-day abstinence from smoking at both the 3-month assessment and the 6-month follow-up assessment is also reported for an efficacy subset of 7726 participants comprising those who provided smoking data at both the 3-month assessment and at the 6-month assessment. Rates of past 30-day point prevalence abstinence from smoking observed in the ITT sample and in the efficacy subset samples were considered as lower and upper bound estimates of the rates of past 30-day point prevalence abstinence from smoking at each follow-up assessment.

Factors associated with past 30-day abstinence from smoking at the 6-month assessment were examined through two logistic regression models, with each model conducted in two steps. In model 1 step 1, six demographic variables (age, sex, race/ethnicity, annual household income, education level, and US census region), four smoking history variables (age of first smoking, lifetime years of regular smoking, number of smoking days in the 30 days prior to the baseline assessment, number of cigarettes smoked per day in the 30 days prior to the baseline assessment), one e-cigarette use variable (past 30-day use of a secondary e-cigarette), and four JUUL use variables (place of first JUUL purchase, number of days of JUUL use in the past 30 days, primary JUULpod flavor used in the past 30 days, and having purchased a JUUL vaporizer to help quit smoking) were entered as predictor variables. To assess the extent to which the effect of participants’ primary use of JUULpod flavors on past 30-day abstinence from smoking at the 6-month assessment was moderated by the place at which participants purchased their first JUUL Starter Kit, an interaction term for “primary JUULpod flavor use”*“place of first purchase of a JUUL Starter Kit” was entered at step 2. Model 2 replicated model 1 with the exception that the variable “primary JUULpod flavor used in the past 30 days” was replaced by the variable “JUULpod flavors used regularly in the past 30 days” (exclusive use of JUUL tobacco flavors vs. exclusive use of JUUL characterizing flavors vs. use of flavors from both categories).

Odds ratios are reported unadjusted and adjusted for the effects of other variables in the model. Odds ratios in these regression models indicate the proportionate change in a participant’s odds of self-reporting past 30-day abstinence from smoking associated with the indicator on the categorical predictor variable. Analyses were conducted using SPSS, v. 25.0. *p* values < 0.05 were considered statistically significant.

## Results

### Past 30-day point prevalence abstinence from cigarette smoking

#### ITT sample

**Fig. 1:**
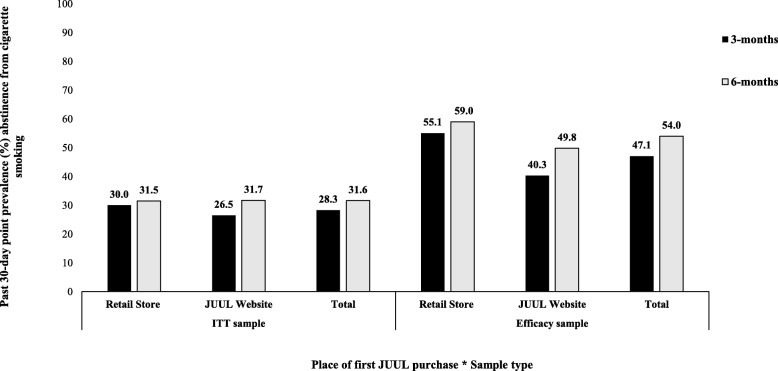
Self-reported past 30-day point prevalence abstinence from smoking assessed at 3 months and 6 months after first purchase of a JUUL Starter Kit, stratified by place of first purchase of a JUUL Starter Kit and sample type

**Fig. 2:**
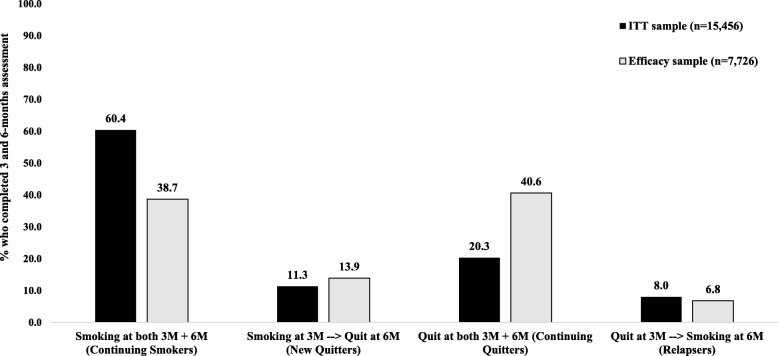
Transitions in cigarette smoking status between the 3-month assessment and the 6-month assessment among new users of a JUUL vaporizer who completed both assessments

In the ITT sample (*N* = 15,456), past 30-day point prevalence abstinence from smoking increased from 28.3% at the 3-month assessment to 31.6% at the 6-month assessment (Fig. 1). Approximately 20.3% of the ITT sample reported past 30-day smoking abstinence at both the 3-month assessment and the 6-month assessment (i.e., continuing quitters) (Fig. 2). In contrast, 60.4% of the ITT sample did not report past 30-day smoking abstinence at either the 3-month assessment or the 6-month assessments (i.e., continuing smokers). The rate at which participants quit smoking between the third and sixth months (11.3%) was approximately 1.4 times higher than the rate at which participants lapsed back to smoking between the third and sixth (8.0%).

#### Efficacy subset samples

When the analysis was restricted to only participants who completed the 3-month assessment (*n* = 9272), self-reported past 30-day point prevalence abstinence from smoking was 47.1%, with the abstinence rate higher among initial retail purchasers (55.1%; *n* = 2346/4260) than among initial online purchasers (40.3%; *n* = 2021/5012). When the analysis was restricted to only those who completed the 6-month assessment (*n* = 9040), past 30-day point prevalence abstinence from smoking was 54.0% (4886/9040), with the abstinence rate again higher among initial retail purchasers (59.0%; *n* = 2467/4180) than among initial online purchasers (49.8%; *n* = 2419/4860).

Last, when the analysis was restricted to only participants who completed both the 3-month assessment and the 6-month assessment (*n* = 7726), past 30-day point prevalence abstinence from smoking at both the 3-month assessment and the 6-month assessment was 40.6%. In contrast, 38.7% did not report past 30-day smoking abstinence at either the 3-month assessment or the 6-month assessments (i.e., continuing smokers). The rate at which participants became abstinent at the 6-month assessment having been smoking at the 3-month assessment (i.e., quit smoking between the third and sixth months, 13.9%) was approximately 2.0 times higher than the rate at which participants who were abstinent from smoking at the 3-month assessment returned to smoking at the 6-month assessment (i.e., lapsed to smoking between the third and sixth months, 6.8%).

### Factors associated with past 30-day smoking abstinence at 6 months

#### Model 1

Demographic, cigarette smoking, and e-cigarette use characteristics of new purchasers of a JUUL Starter Kit who completed the 6-month follow-up assessment (*n* = 9040) are summarized in Table 1, stratified by smoking abstinence status at the 6-month assessment. Adjusting for the effects of all other variables in the model, participants’ odds of reporting past 30-day abstinence from smoking at the 6-month assessments significantly varied by five JUUL-related variables—(i) primary JUULpod flavor used in the 30 days prior to the 6-month assessment, (ii) number of days of use of a JUUL vaporizer in the 30 days prior to the 6-month assessment, (iii) place of first purchase of a JUUL Starter Kit, (iv) whether or not participants first purchased a JUUL vaporizer to help them quit smoking cigarettes, and (v) past 30-day use of any other e-cigarette other than JUUL vaporizer; three smoking-related variables—(i) number of smoking days in the 30 days prior to the baseline assessment, (ii) number of cigarettes smoked per smoking day at the baseline assessment, and (iii) number of lifetime years of regular smoking; and two demographic variables—(i) education level and (ii) census region (Table 2).

**Table 1:** Characteristics of participants who completed the 6-month follow-up assessment (*n* = 9040; 58.5% of ITT sample), by cigarette smoking status at the 6-month assessment

Variable	Cigarette smoking status at 6-month assessment		
	Smoked in the past 30 days (*n* = 4154) *N* %	No smoking in the past 30 days (*n* = 4886) *N* %	Total (*n* = 9040) *N* %
Demographic variables			
Sex			
Male	2287 (55.1)	2872 (58.8)	5159 (57.1)
Female	1798 (43.3)	1954 (40.0)	3752 (41.5)
Transgender	25 (0.6)	28 (0.6)	53 (0.6)
Missing	44 (1.1)	32 (0.7)	76 (0.8)
Age			
21–24	1145 (27.6)	1846 (37.8)	2991 (33.1)
25–34	1444 (34.8)	1623 (33.2)	3067 (33.9)
35–44	833 (20.1)	746 (15.3)	1579 (17.5)
45–54	443 (10.7)	381 (7.8)	824 (9.1)
55–64	227 (5.5)	261 (5.3)	488 (5.4)
≥ 65	62 (1.5)	29 (0.6)	91 (1.0)
Race/ethnicity			
Non-Hispanic, White	2884 (69.4)	3255 (66.6)	6139 (67.9)
Non-Hispanic, Black	85 (2.0)	120 (2.5)	205 (2.3)
Non-Hispanic, Others^§^	528 (12.7)	597 (12.2)	1125 (12.4)
Hispanic^†^	332 (8.0)	480 (9.8)	812 (9.0)
Missing	325 (7.8)	434 (8.9)	759 (8.4)
Education			
Not HS graduate	111 (2.7)	122 (2.5)	233 (2.6)
GED	151 (3.6)	204 (4.2)	355 (3.9)
HS graduate	625 (15.0)	913 (18.7)	1538 (17.0)
Some college or associate’s degree	1618 (39.0)	1789 (36.6)	3407 (37.7)
Bachelor’s degree or higher	1466 (35.3)	1579 (32.3)	3045 (33.7)
Missing	183 (4.4)	279 (5.7)	462 (5.1)
Household income			
< $25,000	831 (20.0)	1007 (20.6)	1838 (20.3)
$25,000 to $74,999	1692 (40.7)	2027 (41.5)	3719 (41.1)
≥ $75,000	1137 (27.4)	1285 (26.3)	2422 (26.8)
Missing	494 (11.9)	567 (11.6)	1061 (11.7)
US census region			
Northeast	957 (23.0)	1016 (20.8)	1973 (21.8)
South	1502 (36.2)	1812 (37.1)	3314 (36.7)
Midwest	1008 (24.3)	1116 (22.8)	2124 (23.5)
West	647 (15.6)	913 (18.7)	1560 (17.3)
Missing	40 (1.0)	29 (0.6)	69 (0.8)
Smoking and e-cigarette variables			
Age of first smoking			
≤ 11 years	144 (3.5)	155 (3.2)	299 (3.3)
12 to 14 years	1025 (24.7)	888 (18.2)	1913 (21.2)
15 to 17 years	1613 (38.8)	1773 (36.3)	3386 (37.5)
18 to 24 years	1284 (30.9)	1958 (40.1)	3242 (35.9)
≥ 25 years	77 (1.9)	99 (2.0)	176 (1.9)
Missing	11 (0.3)	13 (0.3)	24 (0.3)
Lifetime years of smoking			
≤ 1 year	272 (6.5)	556 (11.4)	828 (9.2)
1–5 years	980 (23.6)	1600 (32.7)	2580 (28.5)
6–10 years	898 (21.6)	1000 (20.5)	1898 (21.0)
11–20 years	1065 (25.6)	923 (18.9)	1988 (22.0)
≥ 20 years	860 (20.7)	692 (14.2)	1552 (17.2)
Missing	79 (1.9)	115 (2.4)	194 (2.1)
Number of smoking days in 30 days prior to baseline			
1–9 days	412 (9.9)	847 (17.3)	1259 (13.9)
10–19 days	380 (9.1)	754 (15.4)	1134 (12.5)
20–29 days	864 (20.8)	1213 (24.8)	2077 (23.0)
30 days	2498 (60.1)	2072 (42.4)	4570 (50.6)
Cigarettes smoked per day at baseline			
1–9 cigarettes per day	1858 (44.7)	2907 (59.5)	4765 (52.7)
10–19 cigarettes per day	1368 (32.9)	1284 (26.3)	2652 (29.3)
≥ 20 cigarettes per day	928 (22.3)	695 (14.2)	1623 (18.0)
Days of JUUL vaporizer use in past 30 days at 6 months			
1–9 days	469 (11.3)	321 (6.6)	790 (8.7)
10–19 days	636 (15.3)	431 (8.8)	1067 (11.8)
20–29 days	748 (18.0)	677 (13.9)	1425 (15.8)
30 days	1960 (47.2)	2994 (61.3)	4954 (54.8)
Missing	341 (8.2)	463 (9.5)	804 (8.9)
Current use of an e-cigarette other than JUUL vaporizer			
Yes	553 (13.3)	483 (9.9)	1036 (11.5)
No	3596 (86.6)	4396 (90.0)	7992 (88.4)
Missing	5 (0.1)	7 (0.1)	12 (0.1)
Place of first JUUL vaporizer purchase			
Retail store	1713 (41.2)	2467 (50.5)	4180 (46.2)
JUUL website	2441 (58.8)	2419 (49.5)	4860 (53.8)
Bought JUUL vaporizer SK “to help me quit smoking”			
Yes	3435 (82.7)	4103 (84.0)	7538 (83.4)
No	719 (17.3)	783 (16.0)	1502 (16.6)
Primary JUULpod flavor used in past 30 days (at 6 months)			
Virginia tobacco	477 (11.5)	423 (8.7)	900 (10.0)
Mint	820 (19.7)	1204 (24.6)	2024 (22.4)
Mango	938 (22.6)	1334 (27.3)	2272 (25.1)
Crème	218 (5.2)	180 (3.7)	398 (4.4)
Fruit	134 (3.2)	127 (2.6)	261 (2.9)
Cucumber	173 (4.2)	169 (3.5)	342 (3.8)
Classic tobacco	116 (2.8)	54 (1.1)	170 (1.9)
Menthol	194 (4.7)	170 (3.5)	364 (4.0)
Equal use of 2+ flavors, no primary	747 (18.0)	766 (15.7)	1513 (16.7)
Missing	337 (8.1)	459 (9.4)	796 (8.8)
JUULpod flavors used in past 30 days (at 6 months)			
Only used JUUL vaporizer tobacco flavors*	443 (10.7)	335 (6.9)	778 (8.6)
Only used JUUL vaporizer characterizing flavors^^^	2741 (66.0)	3531 (72.3)	6272 (69.4)
Used both tobacco and characterizing flavors	637 (15.3)	568 (11.6)	1205 (13.3)
Missing	333 (8.0)	452 (9.3)	785 (8.7)

*Abbreviations*: *SK* JUUL Starter Kit (JUUL vaporizer plus four JUULpods), *HS* high school, *GED* General Educational Development, *PI* Pacific Islander

^§^Includes Asian Indian, Chinese, Filipino, Japanese, Korean, Vietnamese, Guamanian, Chamorro, Samoan, and multiple races

^†^Includes Mexican, Cuban, Puerto Rican, and “other Hispanic” ethnicity

*JUUL vaporizer tobacco flavors include “Virginia tobacco” and “classic tobacco”

^^^JUUL vaporizer characterizing flavors include “mint,” “mango,” “crème,” “fruit,” “cucumber,” and “menthol”

**Table 2:** Factors associated with smokers’ likelihood of reporting past 30-day abstinence from cigarette smoking at the 6-month assessment

		Unadjusted	Model 1 adjusted		Model 2 adjusted	
			Step 1	Step 2	Step 1	Step 2
Predictor variable	% P30A	Unadjusted OR (95% CI)	aOR (95% CI)	aOR (95% CI)	aOR (95% CI)	aOR (95% CI)
Sex						
Male	55.7	Ref.	Ref.	Ref.	Ref.	Ref.
Female	52.1	0.87 (0.80–0.94)**	0.96 (0.86–1.07)	0.96 (0.86–1.08)	0.95 (0.85–1.06)	0.95 (0.85–1.06)
Transgender	52.8	0.89 (0.52–1.53)	0.82 (0.42–1.59)	0.82 (0.42–1.59)	0.87 (0.45–1.67)	0.87 (0.45–1.67)
Age						
21–24	61.7	Ref.	Ref.	Ref.	Ref.	Ref.
25–34	52.9	0.70 (0.63–0.77)***	0.96 (0.82–1.12)	0.96 (0.82–1.12)	0.96 (0.82–1.13)	0.96 (0.82–1.13)
35–44	47.2	0.56 (0.49–0.63)***	1.00 (0.79–1.25)	1.00 (0.80–1.25)	1.00 (0.80–1.25)	1.00 (0.80–1.25)
45–54	46.2	0.53 (0.46–0.62)***	1.09 (0.82–1.46)	1.10 (0.83–1.47)	1.10 (0.83–1.46)	1.10 (0.82–1.46)
55–64	53.5	0.71 (0.59–0.87)**	0.9 (0.62–1.3)	0.91 (0.63–1.32)	0.91 (0.63–1.31)	0.91 (0.63–1.31)
≥ 65	31.9	0.29 (0.19–0.45)***	0.72 (0.39–1.36)	0.72 (0.38–1.36)	0.72 (0.38–1.35)	0.72 (0.38–1.34)
Race/ethnicity						
Non-Hispanic, White alone	53.0	Ref.	Ref.	Ref.	Ref.	Ref.
Non-Hispanic, Black alone	58.5	1.25 (0.94–1.66)	1.38 (0.97–1.95)	1.38 (0.97–1.96)	1.35 (0.96–1.92)	1.36 (0.96–1.92)
Non-Hispanic, Others^§^	53.1	1.00 (0.88–1.14)	0.95 (0.81–1.11)	0.95 (0.81–1.11)	0.96 (0.82–1.12)	0.96 (0.82–1.12)
Hispanic^†^	59.1	1.28 (1.10–1.49)**	1.08 (0.90–1.29)	1.07 (0.90–1.29)	1.07 (0.89–1.28)	1.06 (0.89–1.28)
Education						
Not HS graduate	52.4	Ref.	Ref.	Ref.	Ref.	Ref.
GED	57.5	1.23 (0.88–1.71)	1.55 (1.03–2.34)*	1.56 (1.03–2.35)*	1.58 (1.05–2.37)*	1.58 (1.05–2.38)*
HS graduate	59.4	1.33 (1.01–1.75)*	1.09 (0.78–1.53)	1.10 (0.79–1.54)	1.10 (0.79–1.54)	1.10 (0.79–1.54)
Some college or associate’s degree	52.5	1.01 (0.77–1.31)	0.96 (0.70–1.33)	0.97 (0.70–1.33)	0.96 (0.70–1.33)	0.97 (0.70–1.33)
Bachelor’s degree or higher	51.9	0.98 (0.75–1.28)	0.90 (0.65–1.25)	0.90 (0.65–1.26)	0.92 (0.66–1.27)	0.92 (0.66–1.27)
Household income						
< $25,000	54.8	Ref.	Ref.	Ref.	Ref.	Ref.
$25,000 to $74,999	54.5	0.99 (0.88–1.11)	1.07 (0.93–1.23)	1.07 (0.93–1.23)	1.08 (0.94–1.24)	1.08 (0.94–1.24)
≥ $75,000	53.1	0.93 (0.83–1.05)	1.10 (0.94–1.29)	1.10 (0.94–1.29)	1.12 (0.95–1.30)	1.12 (0.95–1.31)
US census region						
Northeast	51.5	0.75 (0.66–0.86)***	0.84 (0.71–0.99)*	0.83 (0.70–0.99)*	0.84 (0.71–1.00)*	0.84 (0.71–1.00)*
South	54.7	0.85 (0.76–0.97)*	0.97 (0.83–1.13)	0.96 (0.83–1.13)	0.97 (0.83–1.13)	0.97 (0.83–1.13)
Midwest	52.5	0.78 (0.69–0.90)***	0.84 (0.71–0.99)*	0.84 (0.71–0.99)*	0.85 (0.72–1.00)	0.85 (0.72–1.00)
West	58.5	Ref.	Ref.	Ref.	Ref.	Ref.
Age of first smoking						
≤ 11 years	51.8	Ref.	Ref.	Ref.	Ref.	Ref.
12 to 14 years	46.4	0.80 (0.63–1.03)	0.74 (0.54–1.00)	0.73 (0.54–1.00)*	0.74 (0.55–1.01)	0.74 (0.55–1.01)
15 to 17 years	52.4	1.02 (0.81–1.29)	0.80 (0.59–1.07)	0.79 (0.59–1.07)	0.81 (0.60–1.08)	0.80 (0.60–1.08)
18 to 24 years	60.4	1.42 (1.12–1.80)**	0.91 (0.67–1.23)	0.90 (0.66–1.22)	0.91 (0.67–1.24)	0.91 (0.67–1.24)
≥ 25 years	56.3	1.19 (0.82–1.74)	0.89 (0.53–1.48)	0.89 (0.53–1.50)	0.86 (0.52–1.44)	0.86 (0.52–1.44)
Lifetime years of smoking						
≤ 1 year	67.1	2.54 (2.13–3.03)***	1.99 (1.44–2.77)***	2.00 (1.44–2.77)***	2.00 (1.44–2.77)***	2.00 (1.44–2.77)***
1–5 years	62.0	2.03 (1.79–2.31)***	1.62 (1.23–2.13)**	1.63 (1.24–2.14)**	1.63 (1.24–2.14)***	1.63 (1.24–2.14)***
6–10 years	52.7	1.38 (1.21–1.58)***	1.27 (0.99–1.63)	1.28 (0.99–1.64)	1.27 (0.99–1.63)	1.27 (0.99–1.62)
11–20 years	46.4	1.08 (0.94–1.23)	1.16 (0.94–1.44)	1.16 (0.94–1.44)	1.16 (0.94–1.43)	1.16 (0.94–1.43)
≥ 20 years	44.6	Ref.	Ref.	Ref.	Ref.	Ref.
Number of smoking days in 30 days prior to baseline						
1–9 days	67.3	2.48 (2.17–2.83)***	2.37 (1.97–2.85)***	2.38 (1.98–2.87)***	2.36 (1.96–2.83)***	2.36 (1.96–2.83)***
10–19 days	66.5	2.39 (2.09–2.74)***	2.15 (1.79–2.57)***	2.15 (1.80–2.58)***	2.15 (1.80–2.58)***	2.15 (1.80–2.58)***
20–29 days	58.4	1.69 (1.52–1.88)***	1.49 (1.30–1.71)***	1.50 (1.30–1.72)***	1.49 (1.30–1.71)***	1.49 (1.30–1.72)***
30 days	45.3	Ref.	Ref.	Ref.	Ref.	Ref.
Cigarettes smoked per day at baseline						
1–9 cigarettes per day	61.0	Ref.	Ref.	Ref.	Ref.	Ref.
10–19 cigarettes per day	48.4	0.60 (0.55–0.66)***	0.87 (0.76–0.99)*	0.87 (0.77–1.00)*	0.87 (0.77–1.00)*	0.87 (0.77–1.00)*
≥ 20 cigarettes per day	42.8	0.48 (0.43–0.54)***	0.78 (0.66–0.92)**	0.78 (0.66–0.92)**	0.79 (0.67–0.93)**	0.79 (0.67–0.93)**
Number of days of JUUL use in past 30 days (at 6 months)						
1–9 days	40.6	0.45 (0.38–0.52)***	0.38 (0.31–0.45)***	0.38 (0.31–0.45)***	0.36 (0.30–0.43)***	0.36 (0.30–0.43)***
10–19 days	40.4	0.44 (0.39–0.51)***	0.39 (0.33–0.46)***	0.39 (0.33–0.46)***	0.38 (0.32–0.44)***	0.38 (0.32–0.44)***
20–29 days	47.5	0.59 (0.53–0.67)***	0.45 (0.39–0.52)***	0.45 (0.39–0.52)***	0.44 (0.38–0.51)***	0.44 (0.38–0.51)***
30 days	60.4	Ref.	Ref.	Ref.	Ref.	Ref.
Current use of an e-cigarette other than JUUL						
Yes	46.6	0.71 (0.63–0.81)***	0.80 (0.67–0.95)*	0.79 (0.67–0.95)*	0.80 (0.67–0.95)*	0.80 (0.67–0.95)*
No	55.0	Ref.	Ref.	Ref.	Ref.	Ref.
Place of first JUUL purchase						
Retail store	59.0	1.45 (1.34–1.58)***	1.22 (1.08–1.37)**	1.24 (0.89–1.74)	1.23 (1.10–1.38)***	1.35 (0.93–1.95)
JUUL website	49.8	Ref.	Ref.	Ref.	Ref.	Ref.
Bought JUUL SK “to help me quit smoking”						
Yes	54.4	1.10 (0.98–1.23)	1.46 (1.26–1.68)***	1.46 (1.26–1.69)***	1.47 (1.27–1.70)***	1.47 (1.27–1.70)***
No	52.1	Ref.	Ref.	Ref.	Ref.	Ref.
Primary JUULpod flavor used in past 30 days (at 6 months)						
Virginia tobacco	47.0	Ref.	Ref.	Ref.	Ref.	Ref.
Mint	59.5	1.66 (1.41–1.94)***	1.45 (1.20–1.76)***	1.51 (1.19–1.91)**	NI	NI
Mango	58.7	1.60 (1.37–1.87)***	1.39 (1.15–1.67)***	1.44 (1.15–1.82)**	NI	NI
Crème	45.2	0.93 (0.73–1.18)	0.96 (0.73–1.27)	1.05 (0.74–1.49)	NI	NI
Fruit	48.7	1.07 (0.81–1.41)	1.30 (0.94–1.81)	1.34 (0.89–2.04)	NI	NI
Cucumber	49.4	1.10 (0.86–1.41)	0.91 (0.68–1.22)	0.80 (0.55–1.15)	NI	NI
Classic tobacco	31.8	0.52 (0.37–0.74)***	0.82 (0.54–1.24)	0.79 (0.48–1.31)	NI	NI
Menthol	46.7	0.99 (0.77–1.26)	1.01 (0.75–1.36)	1.01 (0.70–1.46)	NI	NI
Equal use of 2+ flavors, no primary	50.6	1.16 (0.98–1.36)	1.06 (0.87–1.29)	0.99 (0.77–1.28)	NI	NI
JUULpod flavors used in the past 30 days (at 6 months)						
Only used JUUL tobacco flavors^∆^	43.1	Ref.	NI	NI	Ref.	Ref.
Only used JUUL characterizing flavors^^^	56.3	1.70 (1.47–1.98)***	NI	NI	1.37 (1.14–1.65)**	1.41 (1.13–1.75)**
Used flavors from both tobacco and categories	47.1	1.18 (0.98–1.41)	NI	NI	0.98 (0.79–1.22)	1.03 (0.79–1.35)
Interaction term: primary JUULpod flavor used in past 30 days (at 6 months) × place of first JUUL purchase						
Virginia tobacco × retail				Ref.	NI	NI
Mint × retail	–	–	–	0.92 (0.62–1.37)	NI	NI
Mango × retail	–	–	–	0.91 (0.62–1.35)	NI	NI
Crème × retail	–	–	–	0.78 (0.44–1.40)	NI	NI
Fruit × retail	–	–	–	0.93 (0.48–1.81)	NI	NI
Cucumber × retail	–	–	–	1.42 (0.77–2.62)	NI	NI
Classic tobacco × retail	–	–	–	1.12 (0.45–2.76)	NI	NI
Menthol × retail	–	–	–	0.99 (0.54–1.83)	NI	NI
Equal use of 2+ flavors, no primary × retail	–	–	–	1.13 (0.75–1.70)	NI	NI
Interaction term: JUULpod flavors used in the past 30 days (at 6 months) × place of first JUUL purchase						
Only JUUL tobacco flavors × retail	–	–	NI	NI	–	Ref.
Only JUUL characterizing flavors × retail	–	–	NI	NI	–	0.92 (0.62–1.35)
Both flavor categories and tobacco × retail	–	–	NI	NI	–	0.86 (0.54–1.36)

Model 1: *N* = 6585, *χ*^2^ = 744.631, df = 46, *p <* 0.001

Model 2: *N* = 6591, *χ*^2^ = 727.121, df = 40, *p <* 0.001

*** *p* < 0.001; ** *p* < 0.010; * *p* < 0.050

*Abbreviations*: *P30A* past 30-day abstinence from smoking at the 6-month assessment, *aOR* adjusted odds ratio, *HS* high school, *CPD* cigarettes smoked per day, *PI* Pacific Islander, *NI* not included in the logistic regression model

Unadjusted ORs were estimated using only the relevant variable as the predictor variable

^§^Includes Asian Indian, Chinese, Filipino, Japanese, Korean, Vietnamese, Guamanian, Chamorro, Samoan, and multiple races

^†^Includes Mexican, Cuban, Puerto Rican, and “other Hispanic” ethnicity

^∆^JUUL tobacco flavors include “Virginia tobacco” and “classic tobacco”

^^^JUUL characterizing flavors include “mint,” “mango,” “crème,” “fruit,” “cucumber,” and “menthol”

##### JUUL-related factors

Mint and mango were the most common primary JUULpod flavors used in the past 30 days at 6 months. Past 30-day primary users of mint- and mango-flavored JUULpods together accounted for 51.9% of all participants who had not smoked a cigarette in the 30 days prior to the 6-month assessment, and 47.5% of all participants who completed the 6-month assessment. Compared to those who primarily used Virginia tobacco-flavored JUULpods in the 30 days prior to the 6-month assessment, those who primarily used mint- (aOR = 1.45; 1.20, 1.76) or mango-flavored JUULpods (aOR = 1.39; 1.15, 1.67) were 45% and 39% more likely, respectively, to have not smoked a cigarette in the 30 days prior to the 6-month assessment. Compared to those who primarily used Virginia tobacco-flavored JUULpods, primary users of crème-, fruit-, cucumber-, classic tobacco-, or menthol-flavored JUULpods and those who did not have a primary JUULpod flavor in the 30 days prior to the 6-month assessment had statistically equivalent odds for reporting past 30-day smoking abstinence at the 6-month assessment.

**Fig. 3:**
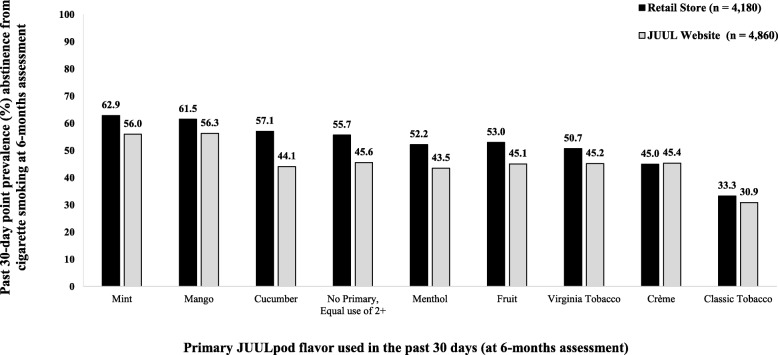
Past 30-day point prevalence self-reported abstinence from cigarette smoking at the 6-month assessment, by primary JUULpod flavor used in the past 30 days and place of first purchase of a JUUL Starter Kit

The interaction term entered at step 2 was not significant, indicating the association between primary JUULpod flavor used in the 30 days prior to the 6-month assessment and past 30-day smoking abstinence at the 6-month assessment was not moderated by the place at which participants purchased their first JUUL Starter Kit. Rates of past 30-day smoking abstinence at the 6-month assessment stratified by past 30-day primary use of JUULpods in eight flavors and place of first purchase of a JUUL Starter Kit, unadjusted for the effects of other variables, summarized in Fig. 3, show that, with the exception of Crème, higher rates of past 30-day smoking abstinence at the 6-month assessment were reported by initial retail purchasers than for all JUULpod flavors.

Compared to those who used a JUUL vaporizer on all 30 of the 30 days prior to the 6-month assessment, those who used a JUUL vaporizer on 20–29 (aOR = 0.45; 0.39, 0.52), 10–19 (aOR = 0.39; 0.33, 0.46), and 1–9 (aOR = 0.38; 0.31, 0.45) of the past 30 days were 2.22 times, 2.56 times, and 2.63 times less likely, respectively, to have not smoked a cigarette in the 30 days prior to the 6-month assessment. Compared to those who indicated they did not purchase their first JUUL Starter Kit “to help to quit smoking cigarettes completely,” those who reported having purchased their first JUUL Starter Kit “to help to quit smoking cigarettes completely” were 46% more likely to have abstained from smoking in the 30 days prior to the 6-month assessment (aOR = 1.46; 1.26, 1.68).

Compared to those who purchased their first JUUL Starter Kit at JUUL’s e-commerce store, those who purchased their first JUUL Starter Kit in a retail store were 22% more likely to have abstained from smoking in the 30 days prior to the 6-month assessment (aOR = 1.22; 1.08, 1.37). Last, compared to those who had not used any e-cigarette other than a JUUL vaporizer in the 30 days prior to the 6-month assessment, those who reported past 30-day use of a secondary e-cigarette were 25% less likely to have abstained from smoking in the 30 days prior to the 6-month assessment (aOR = 0.80; 0.67, 0.95).

##### Cigarette smoking-related factors

Heaviness, frequency, and lifetime duration of cigarette smoking at the time of first purchase of a JUUL Starter Kit were all negatively associated with participants’ odds of reporting past 30-day smoking abstinence at the 6-month assessment. Compared to those who had smoked cigarettes on all 30 of the 30 days prior to the baseline assessment, those who had smoked cigarettes on 20–29 (aOR = 1.49; 1.30, 1.71), 10–19 (aOR = 2.15; 1.79, 2.57), and 1–9 (aOR = 2.37; 1.97, 2.85) of the past 30 days were approximately 1.5 times, 2.2 times, and 2.4 times more likely, respectively, to have not smoked a cigarette in the 30 days prior to the 6-month assessment.

Compared to those who were smoking 1–9 cigarettes per smoking day in the 30 days prior to the baseline assessment, those who were smoking 10–19 cigarettes per day (aOR = 0.87; 0.76, 0.99) and 20 or more cigarettes per day (aOR = 0.78; 0.66, 0.92) were 15% and 28% less likely, respectively, to have not smoked a cigarette in the 30 days prior to the 6-month assessment. Compared to those reported having smoked cigarettes regularly for 20 or more years in their lifetime at the baseline assessment, those who had smoked cigarettes regularly for 0–12 months (aOR = 1.99; 1.44, 2.77) and 1–5 years (aOR = 1.62; 1.23, 2.13) were 99% and 62% more likely, respectively, to have not smoked a cigarette in the 30 days prior to the 6-month assessment.

##### Demographic factors

Compared to those who had not graduated high school, those with a GED were 55% more likely to have abstained from smoking in the 30 days prior to the 6-month assessment (aOR = 1.55; 1.03, 2.34). Compared to those who lived in the West census region, those who lived in the Northeast (aOR = 0.84; 0.71, 0.99) and Midwest (aOR = 0.84; 0.71, 0.99) census regions were 19% and 19% less likely, respectively, to have not smoked a cigarette in the 30 days prior to the 6-month assessment.

#### Model 2

All variables that emerged in model 1 as significant predictors of past 30-day smoking abstinence at the 6-month assessment remained significant in model 2, with no non-significant predictors in model 1 becoming significant in model 2. The added predictor variable—use of JUULpod flavor categories in the 30 days prior to the 6-month assessment—was significantly associated with participants’ odds of reporting past 30-day abstinence from smoking at the 6-month assessment. Compared to those who had exclusively used JUULpods in tobacco flavors in the 30 days prior to the 6-month assessment, those who had exclusively used JUULpods in characterizing flavors were 37% more likely to have abstained from smoking in the 30 days prior to the 6-month assessment (aOR = 1.37; 1.14, 1.65). The interaction term entered at step 2 was non-significant, indicating the association between past 30-day use of JUULpod flavor categories and past 30-day smoking abstinence at the 6-month assessment was not moderated by the place at which participants purchased their first JUUL Starter Kit.

## Discussion

This study prospectively assessed rates of self-reported past 30-day abstinence from cigarette smoking in a large cohort of US adult established current smokers up to 6 months after their first purchase of a JUUL vaporizer. Participants who were still using a JUUL vaporizer and participating in the study after 6 months were typically male, aged 21–34 years, non-Hispanic White, and college-educated, had initiated smoking before aged 18, had smoked cigarettes regularly for fewer than 10 years in their lifetime, and had been daily smokers of 1–9 cigarettes when they purchased their first JUUL Starter Kit. In a worst-case scenario in which non-respondents to survey assessments were assumed to have resumed smoking cigarettes, past 30-day point prevalence abstinence from cigarette smoking was found to have increased from 28.3% at 3 months to 31.6% at 6 months, with 20.3% of participants reporting consecutive past 30-day smoking abstinence outcomes at 3 and 6 months. Additionally, a higher rate of quitting smoking (11.3%) than lapsing to smoking (8.0%) was observed between the third and sixth months of using a JUUL vaporizer.

The impact of the switching from combustible cigarettes to JUUL use on the health of this cohort of adult smokers will depend on the health risks associated with using JUUL products relative to the risks associated with continuing to smoke cigarettes. Though the long-term health risks of e-cigarette use are unknown, and likely will not be well characterized for several years, the best available evidence suggests that, under typical conditions of use, the aerosol emitted by e-cigarettes typically contains fewer and lower concentrations of toxicants and carcinogens than are present in smoke from combustible tobacco cigarettes [1, 30, 31]. If switching completely from smoking cigarettes to using JUUL vaping products specifically is demonstrated to reduce adult smokers’ risks for premature mortality and smoking-related disease to levels that are, at a minimum, significantly below the level of risk associated with continuing to smoke cigarettes, and ideally, statistically equivalent to the levels of risk associated with quitting all tobacco and nicotine use, then the observed rates of complete switching from cigarette smoking to use of a JUUL vaporizer would likely represent a substantial improvement in the health status of this cohort.

However, if the health risks of using a JUUL vaporizer are found to be similar to those associated with smoking cigarettes, the observed rates of complete switching and dual use of cigarettes and JUUL products would likely represent increased harm to the health of these adult smokers. Currently, there is insufficient evidence to conclude that switching completely from cigarettes to JUUL products may modify an adult smoker’s risk of developing serious health problems relative to continuing to smoke tobacco. Empirical data that characterize the risk/safety profile of JUUL vapor products relative to combustible cigarettes, other ENDS products, FDA-approved smoking cessation products, and medications, and to stopping use of all tobacco and nicotine products are therefore urgently needed to more accurately position these products on a continuum of health risk, and so inform the extent to which adult smokers who are unwilling or unable to stop consuming nicotine should be advised to switch from smoking conventional cigarettes to using JUUL vaping products.

Present findings give insight to the patterns of cigarette smoking prior to JUUL initiation and to the patterns of use of JUUL products that may facilitate and impede smokers’ efforts to completely switch to using a JUUL vaporizer within 6 months. Consistent with findings from nationally representative surveys, smokers who reported using a JUUL vaporizer daily at the 6-month assessment were more than twice as likely to have not smoked any cigarettes in those 30 days compared to those who used a JUUL vaporizer less than daily. The use of JUULpods containing characterizing flavors was also found to be an important determinant of smokers’ likelihood of having quit smoking at 6 months. Smokers who reported exclusively using JUULpods containing characterizing flavors—mint, mango, crème, fruit, classic menthol, and/or cucumber—in the 30 days prior to the 6-month assessment were around 38% more likely to have abstained from smoking in those 30 days compared to those who used only JUULpods containing tobacco flavors—Virginia tobacco and/or classic tobacco. Mint or mango was the most commonly used JUULpod flavors at 6 months, and smokers who reported mint and mango as their primary JUULpod flavor choice in the 30 days prior to 6 months were around 46% and 40% more likely to have quit smoking, respectively, compared to those who had primarily used Virginia tobacco-flavored JUULpods. For context, a previous regression analysis found past 30-day primary users of mint- and mango-flavored JUULpods were 37% and 26% more likely than primary users of Virginia tobacco-flavored JUULpods to be past 30-day abstinent from smoking at 3 months [29].. Present findings therefore suggest the associations between past 30-day primary use of mint- and mango-flavored JUULpods and past 30-day smoking abstinence strengthened between the third and sixth months of JUUL use.

The debate about the ratio at which flavored e-cigarettes contribute both to smoking cessation and to tobacco use initiation among youth and non-users at the population level has become increasingly polarized. The FDA has statutory authority over the regulation of flavored e-cigarettes, but any action the agency decides to take must, in the words of Former Commissioner, Dr. Scott Gottlieb, strike a careful public health balance between maintaining access to potentially less harmful sources of nicotine through e-cigarettes for adults who want to transition away from combustible cigarettes, while reducing youth appeal and access to e-cigarettes. A recent editorial in the *New England Journal of Medicine* [32] suggested “the FDA should simply ban the sale of flavored nicotine products for use in e-cigarettes. The public health problem that e-cigarettes can help solve – by helping people who are users of combustible tobacco products stop smoking by switching to vaping – is adequately addressed by liquids that are not flavored to appeal to adolescents.” On this basis, the authors urged the FDA “to use its statutory powers in regulating nicotine delivery devices to take the bold step of removing these flavored products from the market.”

The premise for the authors’ arguing for a complete ban on flavored e-cigarettes is their contention that e-cigarettes flavored to taste like tobacco should be sufficiently appealing and effective to assist adult smokers to transition away from smoking cigarettes. It should be stressed that the present findings neither support the argument that access to JUULpods containing non-tobacco flavors is *essential* for adult smokers to completely substitute a JUUL vaporizer for conventional cigarettes, nor do they rule out the possibility that adult smokers would simply switch to using available tobacco-flavored e-cigarettes in the event that their preferred non-tobacco-flavored products were banned. However, the present finding of a significantly higher odds of quitting smoking associated with the use of non-tobacco-flavored JUULpods contradicts would suggest the opposite hypothesis is more likely to be true: restricting adult smokers’ access to only JUULpods containing tobacco flavors may have the effect of diminishing adult smokers’ openness to try using a JUUL vaporizer as an alternative to continuing to smoke cigarettes and may reduce the likelihood that adult smokers who do begin using a JUUL vaporizer will attempt to quit or cut down smoking and succeed in those attempts.

Investigation of this potential negative effect has become more urgent since JUUL Labs Inc. announced, on 13 November 2018, the company would immediately stop selling its mango-, crème-, fruit-, and cucumber-flavored refill pods to the over 90,000 retail stores in the USA that currently sell JUUL’s flavored refill pods, including convenience stores and specialty vape stores. This action was taken in response to concern expressed by the FDA about the role of non-tobacco flavors in increasing the appeal of vaping to youth, JUUL Labs Inc. Given the current evidence of significantly higher smoking abstinence rates among users of JUULpods containing characterizing flavors and among those who purchased their first JUUL vaporizer in a retail store, the impact of this voluntary action on smoking cessation rates among users of the JUUL vaporizer should be closely assessed. The 6-month data reported here, collection of which concluded on 3 January 2019, may serve as an important baseline for evaluating change in smoking abstinence and relapse rates among users of JUUL products during the period following the removal of flavored JUULpods from retail stores. The collection of data at 12 months will usefully address these questions.

If a statutory ban or voluntary suspension of flavored JUUL products from retail stores were to depress smoking cessation among adults, such actions could then only be justified from a public health perspective if the health benefits that are projected to be lost through a reduction in smoking cessation are outweighed by the health benefits that are projected to be gained from a reduced rate of youth initiation of e-cigarette and other tobacco use attributable to the banning of flavored e-cigarettes. Estimating the impact of JUUL vapor products on the health of the whole population therefore requires collection of data on the use of JUULpods in non-tobacco flavors by youth and non-smokers and the impact of use of these products on these individuals’ use of other tobacco products that may carry more harm, such as conventional cigarettes, to give context to present data that show a significantly higher rate of smoking cessation at 6 months among adults who primarily or regularly use JUULpods in characterizing flavors.

The conclusions of this study are limited in several ways. Smoking abstinence at 3 and 6 months was self-reported, and biochemical verification of self-reported abstinence was not possible due to the large sample size and remote collection of data. Relatedly, it was not possible to estimate change in participants’ overall nicotine consumption associated with their substitution of combustible cigarettes for JUULpods. Though participants were incentivized with $30 to complete each survey, 41.5% of smokers were lost to follow-up at 6 months. Though the study invitation and informed consent procedure conveyed no expectation or requirement that smokers quit smoking in the next 6 months, some may have been unwilling to continue to respond to surveys if they felt embarrassed about not having quit smoking after 6 months. Others may have simply not wanted to continue to provide data after they had quit smoking. Recoding missing respondents as active smokers therefore represents the worst-case estimate of the effect of using JUUL products, but may not represent a realistic estimate. Abstinence rates reported for all enrolled participants and for only those who responded to surveys at 3 and 6 months should therefore be interpreted as lower and upper bound estimates of the effect of using JUUL products on smoking abstinence. Another possible reason for drop-out was that the survey web-link sent to participants by email at the 3 and 6 months was only active for a 10-day window. The researchers received many emails from participants who had not completed their survey before the web-link expired and asked to be sent a new web-link. These requests were rejected in order to maintain a strict measure of change in patterns of cigarette smoking and JUUL use at each assessment that was tied to the time of completion of the participant’s baseline assessment.

Present findings are likely to be valid for adult smokers in the USA who purchase a JUUL Starter Kit of their own volition and use a JUUL vaporizer ad libitum. Findings are less likely to be generalizable to the US adult population of smokers or e-cigarette users, adult smokers who use other brands of e-cigarette, adult smokers who use a JUUL vaporizer for reasons other than to support an attempt to quit smoking, and adult smokers who are less motivated to use e-cigarettes, such as those randomized to receive e-cigarettes or other alternative nicotine products as part of a clinical trial. Several characteristics of the current study design should also caution readers against directly comparing quit rates observed in this study to those reported from randomized controlled trials of e-cigarettes and other nicotine replacement products for smoking cessation. In this study, participants were adults who had purchased JUUL products naturalistically and were recruited opportunistically, observed remotely with no contact from the study investigators, and given no guidance or instruction as to how they should or should not use their JUUL vaporizer. Such naturalistic real-world conditions of product purchase and use are not typical of RCTs of nicotine replacement products. Directly comparing the quit rates observed in a cohort of naturalistic JUUL users against quit rates observed in a trial characterized by tightly controlled participant inclusion/exclusion criteria, conditions of participation, pre-specified and homogeneous regimens of product use, and frequent contact with clinical staff, is therefore ill-advised.

Lastly, by including only those who were adult established current smokers at the time of their first purchase of a JUUL Starter Kit, this study additionally does not yield data on the proportion of all new JUUL purchasers who are adults (versus adolescents) or current smokers (versus former smokers and never smokers). In turn, this study yields no data about the rate of smoking initiation and smoking relapse among those who were not actively smoking or had never smoked a cigarette, respectively, when they purchased their first JUUL vaporizer. Estimating these rates is essential for modeling the impact of using JUUL vapor products on the health of the whole US population, the majority of whom are non-users of tobacco products.

## Conclusions

This study provides evidence of the rate at which a cohort of adult smokers in the USA had completely quit smoking cigarettes 6 months after purchasing a JUUL vaporizer. Around one third of enrolled smokers and one half of smokers who responded to a 6-month follow-up reported being past 30-day abstinent from cigarette smoking after using a JUUL vaporizer for 6 months. The study also identified patterns of use of JUUL products and conventional cigarettes that increased and decreased smokers’ likelihood of having quit smoking at 6 months. More frequent use of a JUUL vaporizer and primary use of JUULpods in characterizing flavors, particularly mint and mango, appeared to be important to smokers’ chances of quitting. The impact of suspending retail sales of flavored JUULpods on adult smokers’ likelihood of quitting should be closely assessed.

## Data Availability

The de-identified datasets analyzed in the current study may be made available to researchers who submit a proposal that is approved by the principal investigator.
